# Hydration and Hardening Properties of High Fly-Ash Content Gel Material for Cemented Paste Backfill Utilization

**DOI:** 10.3390/gels10100623

**Published:** 2024-09-27

**Authors:** Bolin Xiao, Jiandong Wang, Aixiang Wu, Ruiming Guo

**Affiliations:** 1 State Key Laboratory of High-Efficient Mining and Safety of Metal Mine Ministry of Education, University of Science and Technology Beijing, Beijing 100083, China; bxiao@ustb.edu.cn (B.X.); b2067916@ustb.edu.cn (A.W.); grm220523@163.com (R.G.); 2 School of Civil and Resources Engineering, University of Science and Technology Beijing, Beijing 100083, China; 3 Key Laboratory of Safe and Green Mining of Metal Mines with Cemented Paste Backfill of the National Mine Safety Administration, University of Science and Technology Beijing, Beijing 100083, China

**Keywords:** cemented paste backfill, fly ash, gel material, hydration properties, hardening properties

## Abstract

As more and more mines utilize the cemented paste backfill (CPB) mining method, the demand for reducing backfill cost and carbon footprint is increasing and becoming more critical. In this work, a new backfill gel binder made with 40 wt.% of low-quality Class F fly ash (FCM) is proposed to replace ordinary Portland cement (OPC). The binder hydration and gel hardening properties were experimentally investigated through X-ray diffraction, Mercury intrusion porosimetry, uniaxial compression, and thermogravimetric analysis. Three different mine tailings were used to verify the FCM’s applicability. Results show that the strength performance of FCM-CPB is 72% of that of OPC-CPB, while FCM production cost is almost less than half of OPC. The hydration process of the FCM-CPB can be divided into five stages, and the main hydration products are ettringite and gel-like hydrates. The 31.2% porosity of FCM-CPB at 28-day curing is higher than that of 7-day curing, while the average pore size is lower, and the structure is denser. The FCM can meet the strength requirement of three different mine tailings regarding different subsequent filling and cut-and-fill mining methods. The proposed FCM provides a feasible alternative with economic and environmental benefits.

## 1. Introduction

Cemented paste backfill (CPB) uses tailings, cement, and water to produce a homogeneous paste slurry to be piped into underground voids. It is one of the most green and sustainable modern mining solutions, regarding mining accompanying environmental problems such as solid waste surface storage, tailings dam failure, mined-out area collapse, and water contamination [1]. Other benefits, namely providing a safe working platform and increasing recovery rate, are obtained. CPB technology has been applied worldwide for decades [2].

The relatively high cost, mainly the gel material, is one of the outstanding problems restricting CPB utilization [3]. For example, Portland cement can be insufficient and expensive in Northwest China, requiring long-distance road transportation. Furthermore, the annual carbon emissions from cement production are 4 billion tons, making it the world’s second-largest source of industrial carbon dioxide emissions [4]. To tackle these problems, many researchers use solid waste materials such as blast furnace slag, fly ash, and steel slag to produce supplementary cementing material (SCM) and new-type cement to reduce cost and carbon emissions. Fly ash (FA), which is a by-product of thermal power plant combustion dust, is one the massive pozzolanic solid wastes that are generally used as SCM [5].

The utilization of fly ash in the cement industry has been investigated. Elnaz Khankhaje et al. showed that ash’s filler character and pozzolanic activity, which can be alkali-activated to create secondary C–S–H gels, make it an ideal supplementary material in the cement industry [6]. Chuen-Ul Juang used FA as an SCM in cement and found that when 20% of the cement was replaced with fly ash, the concrete strength could be improved [7]. The review showed that the optimum substitution level of cement with FA is 10–30%; however, increasing the replacement level affects the hydration procedure, resulting in lower strength [6]. In the mine backfill area, Tong Zhao et al. prepared a clinker-free slag-based cemented backfill material containing fly ash (10–20 wt.%) that exhibited excellent filling performance [8]. The new backfill binder material with slag-based solid waste can significantly reduce the backfill cost by 30% [3]. The quality of fly ash is essential in utilization, TiO_2,_ and chloride content can serve as an indicator of its reactivity [9]. Jia Li et al. further found that fly ash and gypsum can increase the basicity and activity of fly ash–slag-based binder for mine backfill, obtaining a stable strength growth at the long-term age [10]. Jianxin Fu et al. found that FA affects the internal hydration process and pore evolution of CPB, where excessive FA proportion leads to reduced gel products, increased surface roughness, high porosity, declined strength, and enhanced ductility [11]. Care should be taken when using FA in CPB. Yunhai Cheng et al. replaced 42 wt.% of OPC with FA, obtaining the highest strength of CPB, while a higher FA replacement ratio prolonged the UCS development [12]. Cheng also found that FA had little influence on the pores less than 1 μm, and the UCS gain was not that sensitive to the FA content compared with the cement.

The utilization of FA in CPB is widely studied; however, there is limited research about fly-ash backfill gel material using low-quality FA with an incorporation rate of more than 40 wt.% and replacing backfill cement binder by 100%. In China, there are more than 3 billion tons of low-quality FA (mixed with impurities) stocked on land with an annual production of 550 million tons [13]. and there are billions of cubic meters of underground voids that need to be backfilled. It is meaningful to investigate a new gel material with high fly ash content to replace OPC completely, and to fulfill the potential binder demand of the backfill industry. Barriers are obvious that many backfill binders are developed for specific mine tailings with strict limitations in promotion. Therefore, in this research, a new fly-ash backfill binder is proposed using 40% of low-quality Class F fly-ash. The hydration and hardening properties are investigated. Furthermore, the material is verified using three different mine tailings. The material can reduce backfill costs and carbon emissions significantly.

## 2. Results and Discussion

### 2.1. Strength Properties of the FCM-CPB

**Figure 1:**
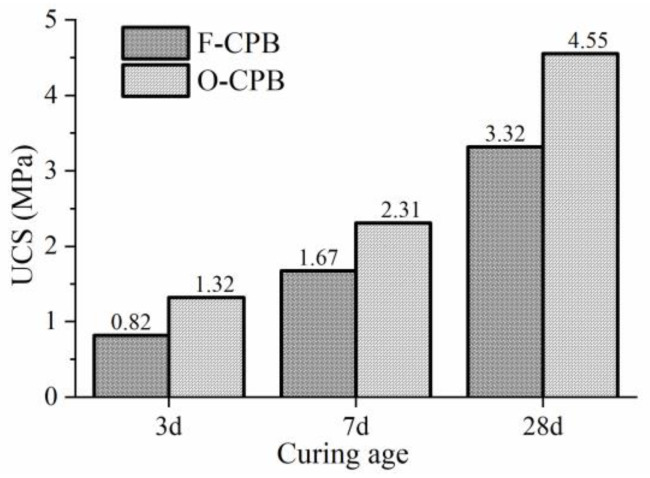
Strength comparison of CPB prepared from two types of gel materials.

The strength results of OPC-CPB and FCM-CPB at the same B/T ratio (1:4) and mass concentration (74 wt.%) are shown in Figure 1. The 3 d, 7 d, and 28 d strengths of FCM-CPB are 0.82 MPa, 1.67 MPa, and 3.32 MPa, respectively. The 3 d, 7 d, and 28 d strengths of OPC-CPB are 1.32 MPa, 2.13 MPa, and 4.55 MPa, respectively. Overall, the strength performance of FCM-CPB is 72% of that of OPC-CPB, regarding its production cost is almost less than half of OPC.Moreover, the production of FCM does not require calcination, which greatly reduces carbon emissions. Therefore, the FCM has significant economic and environmental importance.

Regarding the strength of the CPB, typically, 3 MPa can meet the needs of most mining operations [14]. From engineering experience and design codes [15,16], a CPB body for equipment working platform and second-step mining support pillars requires a 28-day strength of 2.5~3.0 MPa. The backfilling area after the second-step mining needs to be slightly cemented or self-supporting, requiring a 28-day strength of 0.5~1.0 MPa [17]. Therefore, this FCM can meet the needs of most underground mines. In the next part, the applicability of FCM for other mine tailings will be verified.

### 2.2. Hydration Properties of FCM

**Figure 2:**
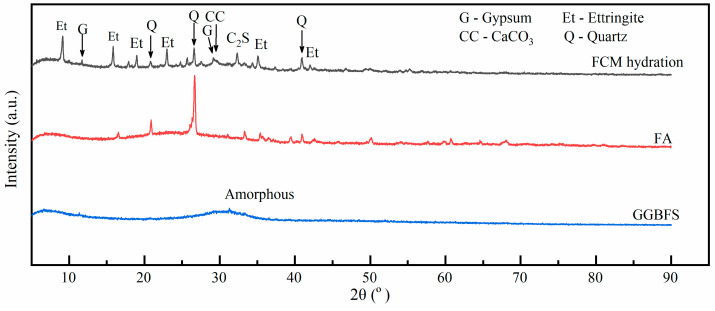
XRD pattern of hydration products of the FCM.

The FCM has a good binding effect on whole tailings. The fundamental reason is the hydration reaction effect of the gel material itself. As time develops, a continuous stream of hydration products is generated and wrapped around tailings’ particles, forming a dense and hard composite structure with the ability to resist loads. The XRD patterns of the hydration products of FCM are shown in Figure 2.

The main mineral components of fly ash include quartz, plagioclase, etc., while slag has mostly irregular amorphous contents. The hydration products of FCM are relatively abundant, mainly including ettringite (AFt), gypsum, calcium carbonate, and some unhydrated C2S, quartz, etc. The other kinds of amorphous hydration products are calcium silicate hydrate (C-S-H) and calcium aluminate hydrate (C-A-H) gel, which can not be detected by XRD. The gels are mainly generated by the reaction of active substances of fly ash and slag with calcium hydroxide [18]. Furthermore, some C-A-H can react with gypsum to produce hydrous calcium aluminium sulfate mineral (ettringite), which contributes to the early mechanical strength of the CPB [19].

**Figure 3:**
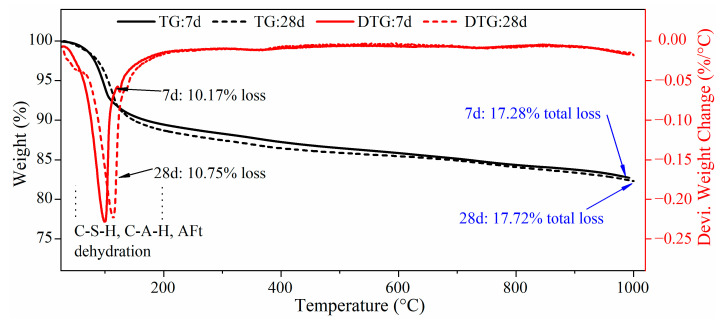
TGA curves of hydration products of FCM.

Together with XRD analysis, the thermogravimetric curve shown in Figure 3 can explore the mass content evolution of these gel-like hydration products.

The dehydration temperature of gel hydrates such as C-S-H is generally between 50~200 °C, which is characterized by a large amount of bound water [20]. Figure 3 shows that this temperature range is the maximum mass loss peak of the whole TGA curve, indicating that the gel-like hydration products have the largest content. Other studies showed that this kind of gel-like product can account for more than 70% of the total hydration products [21]. What needs to be noted is that the dehydration temperature of ettringite is also within the range again. Here, the 7d-FCM hydration product loses 10.17% in this temperature range and increases to 10.75% in 28 days. Other decomposition peak positions of CaOH, CaCO_3_, etc., are not apparent, indicating that the production of such substances is not high. The total mass loss at 1000 °C was 17.72% and 17.28%, respectively, for 7 d and 28 d curing.

The hydration process of FCM-CPB can be summarized into five stages. The first stage: Hydrolyzation of cement clinker, where the main mineral components C2S and C3S hydrolyze to produce a large amount of OH^−^. Stage 2: Slag and fly ash generate H_2_SiO_4_^2−^ and H_2_AlO_3_^−^ under OH^−^ excitation. The reaction rate at this stage is related to the concentration of OH^−^ and Ca^2+^ ions, the particle size of active materials, and the content of amorphous Al^3+^ and Si^4+^. Stage 3: the combination reaction of two ions. One is that Ca^2+^ reacts with dissolved H_2_SiO_4_^2−^ to generate C-S-H gel, and the other is that SO_4_^2−^ dissolved in water reacts with H_2_SiO_4_^2−^ and Ca^2+^ to generate ettringite. Stage 4: A large amount of hydration products, such as C-S-H and AFt, are generated and wrapped around the tailings’ particles. Stage 5: C-S-H and AFt will crystallize on the surface of tailings particles and grow alternately, forming a dense network structure.

### 2.3. Microstructure of the FCM-CPB

**Figure 4:**
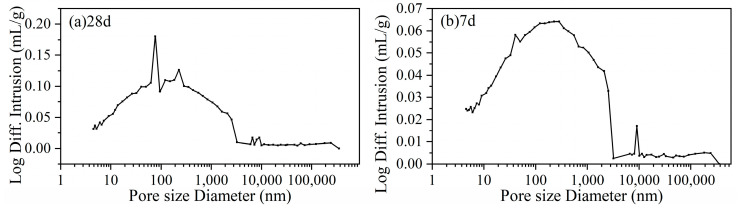
MIP curve of FCM-CPB at (**a**) 28 d and (**b**) 7 d curing.

The strength performance of FCM-CPB is inevitably related to its microscopic pore structure. Its formation is a network structure composed of hydrated products wrapped around tailings’ particles. The pore structure of the CPB at 7 d and 28 d was detected by the mercury intrusion porosimeter, as shown in Figure 4. The pore structure characteristics are shown in Table 1.

The porosity of the 28 d CPB is 31.2%, an increase of 7.4% compared to 7 d. This is because fine active particles remain in the network structure that have not undergone hydration or sedimentation. As hydration proceeds, the active particles in the pores are reacted, the structure is refined, the porosity increases, and the network structure becomes denser. The binder material hydration kinetics, the fineness of the tailings, and the MIP method may play an important role, resulting in a larger porosity of 28 d CPB. This is also consistent with the wider range of pore diameter bands at 28 d and the narrower range of pore diameter bands at 28 d, as shown in Figure 4. The average particle size of the pores (4 V/A) is 99 nm at 28-day curing, which is lower than 101 nm at 7-day curing. The refining effect does not mean filling up the pores but strengthening the stiffness of the structure.

Further research can reveal the relationship between the pore structure characteristics and the mechanical behavior of CPB materials, establish the relationship between micro-scale and macro-scale mechanical properties, and lay the foundation for predicting the performance and computational modeling of CPB materials.

### 2.4. Discussion on Applicability of FCM to Multiple Tailings from 3 Mines

To verify the applicability of the FCM in different practical backfill mines, three different mine tailings were collected to test the strength performance of the CPB. Usually, mine sites prepare CPB with different concentrations and B/T ratios as needed.

#### 2.4.1. Mine Tailings and CPB Parameters

The tailings are taken from three different mines of copper (CT), nickel (NT), and gold (GT) ore processing plants in the northwest region of China. The particle size characteristics of tailings are shown in Table 2. The NT has the finest tailings with 58.55 wt.% of particles below 75 μm. The B/T ratios and concentrations used to prepare CPB are the same as those used in actual mining production.

#### 2.4.2. Strength Verification of FCM

Since UCS is one of the most important properties in CPB application, this section performs UCS tests at different binder contents and solid concentrations to verify the feasibility of FCM in different mine backgrounds.

(1) Copper mine tailings

**Figure 5:**
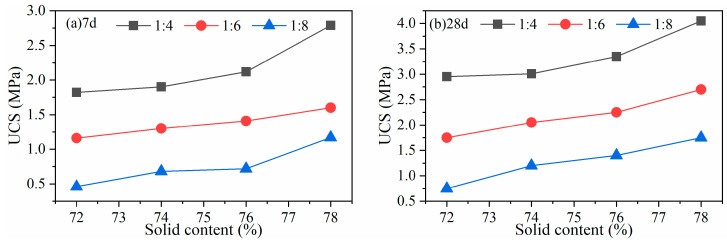
UCS at various binder contents and concentrations for a copper mine at (**a**) 7 d and (**b**) 28 d strength.

The FCM was used to prepare the copper tailings CPB with a B/T ratio from 1:4 to 1:8 and solid mass concentration from 72 to 78 wt.%. The results are shown in Figure 5. When the B/T ratio is 1:6, and the concentration is 78%, the 7-day and 28-day strength of the CPB reaches 1.60 MPa and 2.70 MPa, respectively, meeting the strength requirements for subsequent filling mining method. The 28-day strength at 1:8 is greater than 1.0 MPa, which can be used in the second-stage backfilling for self-support. Therefore, the FCM is technically feasible in this copper mine.

(2) Nickle mine tailings

**Figure 6:**
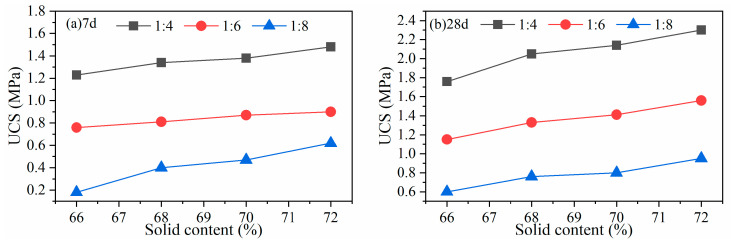
UCS at various binder contents and concentrations for a copper-nickel mine at (**a**) 7 d and (**b**) 28 d strength.

Similar UCS verification was performed using a second nickel mine tailings. The B/T ratio is the same from 1:4 to 1:8; the concentration is set to 66~72% due to the relatively finer tailings. The results shown in Figure 6 indicate that the CPB strength increases approximately linearly with the increase of slurry concentration and B/T ratio. When the B/T ratio is 1:4, the concentration of 72%, the 7-day and 28-day strength are 1.5 MPa (≥1.5 MPa) and 2.30 MPa (>2.00 MPa), respectively, which meet the strength requirements of cut-and-fill stoping mining method in the copper-nickel mine.

(3) Gold mine tailings

**Figure 7:**
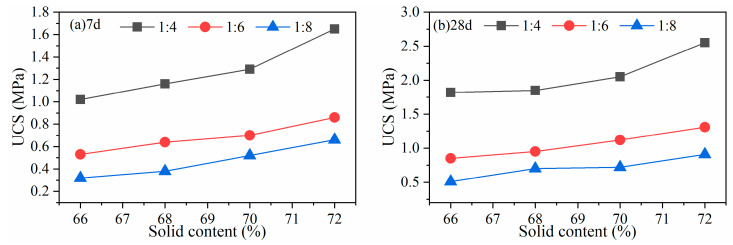
UCS at various binder contents and concentrations for a gold mine at (**a**) 7 d and (**b**) 28 d strength.

A third gold mine tailings was used to verify the FCM feasibility in backfill. The CPB B/T ratio is 1:4~1:8, and the concentrations are 68~74%. The UCS strength results are shown in Figure 7. When the B/T ratio is 1:4 and the slurry concentration is 70%, the 7-day strength of the FCM-CPB reaches 1.21 MPa (>1.0 MPa), and the 28-day strength reaches 2.21 MPa (>2.0 MPa). When the B/T ratio is 1:6 at the same concentration, the 28-day FCM-CPB strength is 1.12 MPa (>1.0 MPa). The strength results in fully meeting the strength requirements of the gold mine utilizing the cut-and-fill mining method.

## 3. Conclusions

This work proposed a high fly ash content gel material for cemented paste backfill. The hydration and hardening properties of the FCM-CPB were investigated. The applicability of the FCM was verified in three different mine backgrounds. The following conclusions can be obtained.

The optimum FCM contains 40 wt.% of fly ash, 10 wt.% of clinker, 12 wt.% of DG, and 38 wt.% of GGBFS, which has 90 wt.% of solid waste. The FCM-CPB shows reasonable mechanical performance, which is 72% of that of OPC-CPB. Considering the use of a large amount of solid waste, low-quality fly ash, and free from sintering, the FCM has outstanding economic and environmental benefits.

The main hydration products of FCM are ettringite and gel-like hydrates, which can account for up to 80%. The hydration process of the FCM-CPB can be divided into five stages. The porosity of CPB at 28 days is higher than that of 7 days, while the average pore size is lower, and the structure is denser.

UCS verification tests at different binder contents and solid concentrations in three copper, nickel, and gold mine backgrounds all proved that the FCM is technically applicable for different mine tailings regarding different mining methods of subsequent filling and cut-and-fill.

## 4. Materials and Methods

### 4.1. Materials

#### 4.1.1. Fly-Ash

**Figure 8:**
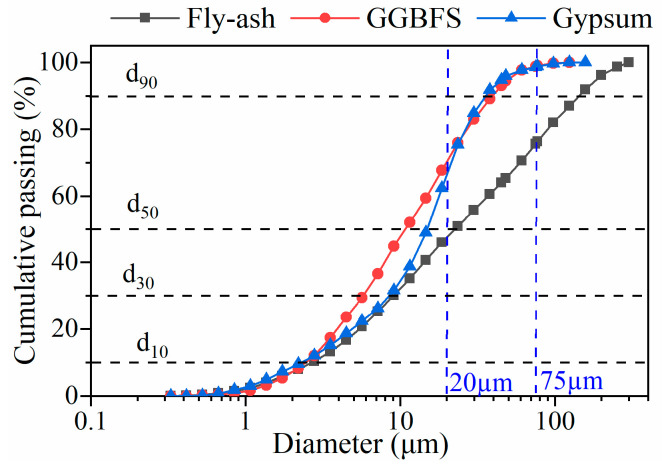
Particle size distribution of the raw material.

There are many thermal power plants in Northwest China, producing large quantities of low-quality fly ash (Class F, low Ca type, and mixed with impurities during output) every year. The fly ash was collected from one of the power plants (Hami, Xinjiang, China) and subjected to a Lazer particle size analyzer for distribution analysis. The result is shown in Figure 8. The chemical properties of the fly ash are shown in Table 3. The specific gravity is 2.25. The total Si, Fe, and Al oxides content accounts for 82.35%, and Ca content accounts for 8.9%, which can be classified as Class F and low Ca type according to ASTM C618 [22].

#### 4.1.2. Desulfurization Gypsum

The desulfurization gypsum (DG) is a byproduct of the power plant’s flue gas desulfurization process. Samples were collected from the same plant. The particle size distribution and the featured particles are shown in Figure 8. The chemical property of DG is listed in Table 3. The CaO and SO_3_ contents account for 44.5% and 41.5%, respectively. It can be calculated that there are about 89% of dihydrate gypsum (CaSO_4_·2H_2_O).

#### 4.1.3. Ground Granulated Blast Furnace Slag

Ground Granulated Blast Furnace Slag (GGBFS) is a byproduct of the iron-making industry and was collected from Da’an Iron and Steel Co., Ltd. (Hami, Xinjiang, China). The specific gravity is 3.28. The reactivity degree is Grade 80, and the surface area is 450 m^2^/kg. The particle size distribution of GGBFS is shown in Figure 1, and the chemical properties are shown in Table 3. When used as a SCM, the reactivity of GGBFS is usually evaluated by alkaline coefficient, quality coefficient, and activity coefficient [23], as shown in Equation (1):

(1) $$\left\{\begin{array}{c} M_{0}=\frac{CaO+MgO}{SiO_{2}+{Al}_{2}O_{3}}\\ K=\frac{CaO+MgO+{Al}_{2}O_{3}}{SiO_{2}+{TiO}_{2}+MnO}\\ M_{a}=\frac{{Al}_{2}O_{3}}{SiO_{2}} \end{array}\right.$$

From the chemical composition in Table 3, the alkaline coefficient *M*_0_ = 1.192 (>1.0), which belongs to alkaline slag and can be used as SCM. The quality coefficient *K* = 1.724 (*K* > 1.6) indicates medium-quality slag that can be used in the cement industry. The activity coefficient *M*_a_ = 0.318 (*M*_a_ > 0.30) indicates it belongs to highly reactive slag. The three indices show that the GGBSF can be utilized in cement production.

#### 4.1.4. Tailings

The tailings were sampled from a tailings dam of a copper mine in Northwest China. The tailings have 6.41 wt.% of fine particles below 20 μm and 23.06 wt.% below 75 μm. The maximum diameter is 650 μm and the medium diameter *D*_50_ = 155.28 μm. The specific gravity of the tailings is 2.74.

#### 4.1.5. The Fly-Ash Cementitious Material (FCM)

**Figure 9:**
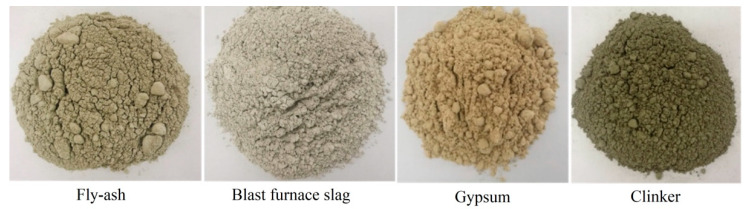
Morphology of the FCM raw material.

This work explores an FCM backfill binder that has a fly ash content of 40 wt.%. The FCM is made of pozzolanic material of fly ash and GGBFS, as well as the activation material of clinker and sulphate. Under substantial previous experiments and experience [24,25]. The earlier work performed uniform experiments on paste strength at various raw material mixing amounts. Then, a quadratic polynomial regression model was established between the paste strength and adding amounts of four raw materials. Thus, the mixing proportion can be obtained by solving the extremum problem of the highest strength. The FCM proportioning was determined through uniform experiments. In the experiment, the fly-ash content was set at 40 wt.%; the clinker content range is from 10 wt.% to 20 wt.%; the DG content range is from 6 wt.% to 16 wt.%; the rest is GGBFS making up to 100 wt.%. The optimum ratio was examined through 7-day paste strength at a mixing water/cement ratio of 0.42. The highest strength group of the binder is composed of 40 wt.% of fly ash, 10 wt.% of clinker, 12 wt.% of DG, and 38 wt.% of GGBFS. The following hydration and CPB strength tests were performed according to this optimum FCM. The appearances of the FCM raw materials are shown in Figure 9.

### 4.2. Experimental Methods

#### 4.2.1. Uniaxial Compression Strength (UCS)

Once the FCM was prepared according to the optimum ratio, natural copper tailings and water were incorporated to prepare the FCM-CPB following the practical binder/tailing (B/T) solid mass ratio of 1:4 and solid mass content of 74 wt.%. Ordinary Portland cement (OPC) was used as a control reference. When preparing the CPB sample (B/T = 1:4, solid mass content = 74 wt.%), the gel material (fly ash/clinker/DG/GGBFS = 40/10/12/38), dry tailings, and water were mixed in a mixer for 5 min obtaining a homogeneous state slurry. Then, the sample was evenly poured into a 7.07 × 7.07 × 7.07 cm triple plastic mold and covered with a plastic film to prevent water evaporation and invasion. Transfer the poured mold to a curing chamber at a constant temperature of 20 ± 1 °C and a relative humidity of 95 ± 1%. After 24 h, the samples were de-molded and placed back in the curing chamber until the desired curing age. A HYE-2000 (Hengyu, Hengshui, China) rigid compression machine was used in the compression test. Before loading, the compression surface area was measured with a vernier caliper, and then the CPB was loaded at a speed of 1 mm/min or 100 N/s until failure. The stress and strain were automatically recorded using the built-in sensors. The UCS is obtained by dividing the peak load by the compression surface area. Each group of samples is tested three times, and the average value is taken as the UCS for repeatability of the result.

#### 4.2.2. Mercury Intrusion Porosimetry (MIP)

The MIP test is used to measure the pore volume and specific surface area of a hardened CPB. Its principle is calculating the pore volume and specific surface area based on the amount of mercury filled in the pores under a certain pressure. According to the Lorentz Mendelssohn equation [26], the amount of mercury filled in the pores is proportional to the pore volume. The MIP test is helpful for studying the strength development and failure characteristics of CPB materials. When preparing the MIP sample, the CPB body was carefully cut into spherical shapes with a diameter of about 10 mm. The mercury intrusion instrument was the AutoPore IV 9500 from Micromeritics Instrument Corporation (Norcross, GA, USA). The measurable pore size range is generally from 0.0064 to 950 μm.

#### 4.2.3. X-ray Diffraction (XRD)

X-ray diffraction is used to analyze the crystalline chemical composition of powder samples. Thus, the mineral types and quantity relationships of the FCM hydration products at different ages can be examined. The paste sample was made with a water/cement ratio of 0.42. Once the desired curing age, the paste sample was oven-dried for 48 h at 60 °C. Then, the dry paste was crushed, ground, and sieved with a 45 μm sieve, obtaining the powder sample. The scanning instrument used a D8 Advance diffractometer from Bruker Corporation (Billerica, MA, USA), with Cu target and Kα rays, at a voltage of 40 kV, a current of 40 mA, a 2θ range of 10~90°, a step size of 0.02°, and a scanning rate of 1 (°)/min.

#### 4.2.4. Thermal Decomposition

The thermal decomposition test investigates the mass change of the sample as temperature increases. Due to the different dehydration and thermal decomposition temperatures of different substances, the mineral composition can be inferred based on the mass loss in different temperature ranges, namely thermogravimetric analysis (TGA) [27]. The TGA here was combined with XRD to perform the semi-quantitative analysis of FCM hydration products at different ages. The TGA experiment used the Q50 thermogravimetric analyzer from TA Instruments (New Castle, DE, United States). The sample used was consistent with the XRD sample. During the experiment, about 10 mg of the powder sample was placed in a nitrogen-protective atmosphere and heated from room temperature to 1000 °C at a rate of 10 °C/min. The instrument can record the relationship curve between mass loss, heat flux, phase transition, etc.

## Figures and Tables

**Table 1:** Pore structure characteristics of FCM-CPB.

Item	Porosity (%)	(Average) 4 V/A (nm)	Intermediate Aperture V (nm)	Intermediate Aperture A (nm)	Vtotal (mL/g)	Total Pore Area (m^2^/g)	Average Pore Volume (mL/g)	Average Pore Area (m^2^/g)
7 d	23.8	101	185.2	13.3	0.148	12.81	0.061	2.44
28 d	31.2	99	153.1	14.5	0.246	21.72	0.094	3.83

**Table 2:** Featured particles of tailings from three different mines.

Type	*D*_10_/μm	*D*_30_/μm	*D*_50_/μm	*D*_60_/μm	*D*_90_/μm	*C*_c_	*C*_u_	−20 μm/%	−75 μm/%
CT	28.04	78.11	155.28	185.35	415.75	1.17	6.61	6.41	23.06
NT	15.62	35.71	62.36	77.12	146.83	1.05	4.94	15.34	58.55
GT	15.13	51.22	83.14	101.66	199.28	1.71	6.72	14.27	45.31

Note: *C*_u_ = *D*_60_/*D*_10_, coefficient of uniformity; *C*_c_ = *D*_30_^2^/(*D*_60_∙*D*_10_), coefficient of curvature; *D_x_* = featured diameter where *x*% of the tailings’ particles pass the featured diameter.

**Table 3:** Chemical composition of the raw material and tailings (wt.%).

Material	CaO	SiO_2_	Al_2_O_3_	MgO	TiO_2_	SO_3_	Fe_2_O_3_	MnO	P_2_O_5_	Others
GGBFS	40.99	32.02	10.19	9.33	2.82	1.82	1.31	0.24	0.01	1.27
DG	44.51	5.68	1.48	4.06	0.10	41.45	1.91	0.02	0.05	0.74
Fly-ash	8.90	56.16	21.02	2.34	1.27	0.57	5.17	/	/	3.57
Clinker	64.69	21.46	4.44	2.89	0.27	0.25	4.69	/	/	1.26
Tailings	28.00	30.37	8.88	7.43	0.34	0.16	22.28	0.44	0.08	2.02

## Data Availability

The original contributions presented in the study are included in the article, further inquiries can be directed to the corresponding authors.
